# Editorial

**Published:** 1877-01

**Authors:** 


### Editorial.

The first completed volume of the Journal and Examiner is now in the hands of its readers, and they can judge for themselves how far the editorial corps has proved itself worthy of the trust conferred upon it by the Chicago Medical Press Association.

Judging from the tenor of many kind letters, and from a gratifying increase in the subscription list, the editors are disposed to believe that the changes made during the year have been approved by the profession. While they are modest enough to accept this compliment, they are not so blinded by a first success as not to appreciate the deficiencies in the character of the year's work.

Upon two of these deficiencies we desire to make a brief comment. It has, from the beginning, been our purpose to make the Journal and Examiner especially useful to the profession of the Northwest. With this view we have introduced the monthly *Summary of Progress of the Medical Sciences* in order to disseminate some knowledge of the observations, researches and doctrines of our colleagues abroad.

But on the other hand the Journal and Examiner should fairly represent the professional workers of the Northwest. Its pages should give, as in a mirror, a truthful representation of the labors of the profession in the district named, by incorporating full reports of the proceedings of its medical societies and hospital clinics. A synopsis of the papers read before the societies, and a brief record of the discussion which may follow the reading of the papers or the presentation of pathological specimens would, by the character of such papers and discussions, show the exact standing of the profession.

And so also the pages of our periodical ought to contain a record of hospital clinics, because the practice and teaching of the physicians and surgeons of the large hospitals may generally be accepted as representing the character of professional practice in a whole district or country. Besides, an accurate and well drawn report of typical cases would be useful and instructive to the readers of the JOURNAL AND EXAMINER.

In both these directions the editors are compelled to say that they have not been able to do justice to the profession. The reports of our medical societies, as furnished by their reporters, have been so deficient that when they finally ceased to appear, neither these pages nor their many readers were great losers.

It was a step in the right direction when the Chicago Medical Society appointed an editor for the publication of the history of cases and pathological specimens presented at its meetings. The second series of these "Pathological Transactions" will be found in this number. It reflects credit upon the editor, the several contributors and the medical society; and indicates that a little enthusiastic effort can render the material which accumulates in the dead records of the societies useful and interesting to the profession at large. If other societies would do likewise the pages of this journal would soon show a splendid record of the scientific zeal of the medical profession in this country.

Still more deplorable have been the reports from our hospitals. A medical journal published in a metropolitan city, like Chicago, provided with large hospitals, is expected to give its readers a fair account of hospital practice. But the editors have, in this respect, to depend on the efficient co-operation of the hospital physicians and surgeons. The latter are to select the cases worth reporting, and must either themselves report them or have the reports written by younger men in the profession. But certainly it should be a matter of interest to the former, one would think, to see that the cases and the comments upon them are correctly reported before they are sent to the editors. The latter cannot be held responsible for the correctness of what is published as the clinical lecture of Dr. X. if Dr. X. does not take pains in selecting an efficient clinical reporter, and in supervising the latter's work. Dr. X. must not be surprised to find his clinical lecture reported in a fashion which he considers a disgrace to himself, if he returned for publication, and without making a single correction, a written report of his lectures which the editors sent him with an urgent request to correct the apparently erroneous statement of his remarks.

The editors are fully conscious of the fact that what clinical reports have been published during the past year very often were imperfect, and the only apology they can offer their readers is, that in the kindness of their hearts they have often appreciated the good will of the writers rather than their capacity as clinical reporters. But the editors are also compelled to say that from the largest hospital in the Northwest they have received neither encouragement nor co-operation; the attending staff of that institution having shown no disposition to furnish reports of their hospital practice. Unless they be, by some miracle, roused from this apparent lethargy, the mass of clinical material furnished by that charity will be buried within its walls; the records of cases will remain a dead letter; and the readers of the JOURNAL AND EXAMINER will not be the wiser for what is said and done within the precincts which are, to the most of them, otherwise inaccessible.

